# Splinkerette PCR for Mapping Transposable Elements in *Drosophila*

**DOI:** 10.1371/journal.pone.0010168

**Published:** 2010-04-13

**Authors:** Christopher J. Potter, Liqun Luo

**Affiliations:** 1 Solomon H. Snyder Department of Neuroscience, Center for Sensory Biology, The Johns Hopkins University School of Medicine, Baltimore, Maryland, United States of America; 2 Howard Hughes Medical Institute, Department of Biology, Stanford University, Stanford, California, United States of America; Katholieke Universiteit Leuven, Belgium

## Abstract

Transposable elements (such as the P-element and piggyBac) have been used to introduce thousands of transgenic constructs into the *Drosophila* genome. These transgenic constructs serve many roles, from assaying gene/cell function, to controlling chromosome arm rearrangement. Knowing the precise genomic insertion site for the transposable element is often desired. This enables identification of genomic enhancer regions trapped by an enhancer trap, identification of the gene mutated by a transposon insertion, or simplifying recombination experiments. The most commonly used transgene mapping method is inverse PCR (iPCR). Although usually effective, limitations with iPCR hinder its ability to isolate flanking genomic DNA in complex genomic loci, such as those that contain natural transposons. Here we report the adaptation of the splinkerette PCR (spPCR) method for the isolation of flanking genomic DNA of any P-element or piggyBac. We report a simple and detailed protocol for spPCR. We use spPCR to 1) map a GAL4 enhancer trap located inside a natural transposon, pinpointing a master regulatory region for olfactory neuron expression in the brain; and 2) map all commonly used centromeric FRT insertion sites. The ease, efficiency, and efficacy of spPCR could make it a favored choice for the mapping of transposable element in *Drosophila*.

## Introduction

The ability to introduce transgenes into an organism has revolutionized biological investigations. This is particularly true for the *Drosophila* model organism. In *Drosophila*, the most commonly used methods for introducing a transgene into the genome is mediated by the P-element transposon [1], [2] or the piggyBac transposon [3]. In these approaches, the transgene to be integrated is flanked by P-element or piggyBac transposable elements ends, which can integrate the transgene into the germline in the presence of a transposase enzyme. The result is a transgene inserted into the genome flanked by transposable element ends.

**Figure 1:**
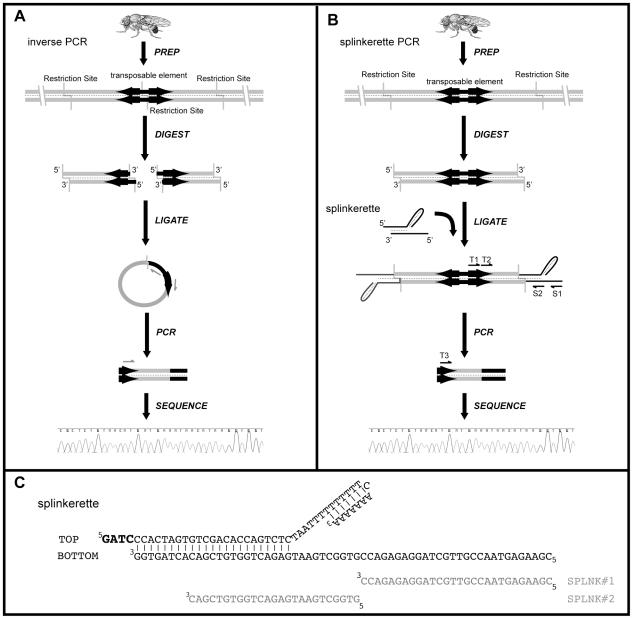
Schematic of PCR methods for mapping transposable elements. **A**) Schematic for the inverse PCR method. Genomic DNA isolated from a fly strain containing a transposable element is digested with an enzyme that cuts within the transposon. These fragments are circularized by a ligation reaction. A PCR reaction with primers designed to the transposon end and an internal sequence amplifies the flanking genomic region. This PCR product is sequenced by a nested primer. **B**) Schematic for the splinkerette PCR method. Genomic DNA is isolated from the fly line containing the transposable element to be mapped. The genomic DNA is digested by an appropriate enzyme that produces sticky ends. The enzyme could cut within the transposable element (similar to scheme A for iPCR) but such digestion is not necessary for the splinkerette PCR reaction. A double stranded splinkerette oligonucleotide with a stable hairpin loop and compatible sticky ends is ligated to the digested genomic DNA. This is followed by two rounds of nested PCR (‘S1’ and ‘T1’ indicate the primer pairs for the first round from splinkerette and transposon, and ‘S2’ and ‘T2’ indicate the primer pairs for the second round of PCR). This generates a PCR fragment that contains the flanking genomic DNA between the transposable element insertion site and the genomic digestion site. A third nested primer directed against the transposon (T3) is then used for a standard Sanger sequencing reaction. In this schematic, only one end of the transposable element is targeted for isolation of flanking genomic DNA. The other end can also be targeted by using different ‘T’ primer pairs specific to this other end. **C**) The annealed splinkerette oligonucleotide sequence is shown along with alignment of the PCR primers SPLNK#1 (S1) and SPLNK#2 (S2). The GATC sticky end is bolded.

It is often useful to determine the exact genomic insertion site for the transgene. Most commonly, this is used for determining 1) which gene is mutated by the insertion; 2) the enhancer regions captured by a particular enhancer trap; 3) which chromosomal segments might be rearranged by a particular insertion such as an FRT site; 4) which gene might be overexpressed by an inserted regulatory element; or 5) the location of a transgene for recombination with other genetic elements, such as a mutation or other transgenes. Since transposon integrated transgenes are derived from cloned sequences, they contain known sequences, which can be utilized to molecularly determine their insertion sites. The most commonly used method for mapping transgene insertion sites is iPCR [4], [5] and plasmid rescue [6]. In the iPCR method (Figure 1A), genomic DNA containing the inserted transposable element is digested with a restriction enzyme that must also cut within the cloned transposable element. This generates a restriction site within the transposon transgene as well as within the neighboring genomic DNA. This transposon-genomic DNA fragment is then ligated back to itself to form a circular DNA structure. By using carefully selected PCR primers which align to the transposon, the genomic fragment is amplified (Figure 1A) and then sequenced. Plasmid rescue is a similar strategy in which the circularized transposon-genomic DNA must contain an origin of replication and a drug resistance marker, which is then isolated after transforming into bacteria for sequence analysis.

The iPCR method is sufficient to map the insertion site for most transposable elements, and has been used in high-throughput screens to isolate the insertion sites for thousands of P-element and piggyBac transposable elements (e.g. Ref. [7]). A major limitation is that the transposable element must be digestible by the restriction enzyme used for iPCR. If not, the transgenic insert, which is often greater than 10 kb, will also be included in the iPCR reaction along with additional flanking genomic DNA. Such large fragments are difficult to PCR amplify. This usually limits the choice of restriction enzymes to four-base pair cutters (BfuCI, HinPI, MspI) that cut close to the transposon end, and in turn limits the size of the genomic DNA that can be isolated. This is problematic when the transgenic transposon insertion is inside a natural transposon, immobilized transposable elements that are present at multiple copies within the genome. In fact, 3.8% of the *Drosophila* genome consists of natural transposons [8] with a 4.7 fold increase in natural transposon density near centromeric regions. The restriction sites commonly used for iPCR often cut within these natural transposons, and so the iPCR method may not be able to isolate genomic DNA that is beyond these natural transposons.

An alternative approach for mapping insertion sites is spPCR (Figure 1B). This technique was originally developed to amplify the genomic DNA between a known restriction site and a target gene [9], and then adapted to map the insertion sites of viral integrating gene traps in the mouse genome [10]. In this technique, genomic DNA is digested to yield overhanging sticky ends (Figure 1B). The restriction enzyme is not required to cut within the transgene. Onto this sticky end is ligated a double stranded oligonucleotide (the splinkerette) that 1) contains a compatible sticky end, 2) contains a stable hairpin loop, and 3) is unphosphorylated (Figure 1C). Two rounds of nested PCR are then performed to amplify the genomic sequence between the transposon insertion and the annealed splinkerette. This is followed by a sequencing reaction with another nested primer. The spPCR reaction remains highly efficient and specific due to the splinkerette design. Since the splinkerette oligonucleotide is not phosphorylated at its 5′ sticky end, only the bottom 3′ recessed strand of the splinkerette sticky end is ligated to the 5′ phosphorylated sticky end of digested genomic DNA. In addition, the PCR primer (‘S1’ in Figure 1B) which anneals to the splinkerette only amplifies DNA that has been generated as a result of a successful first strand synthesis. As a result, the PCR reaction occurs preferentially between genomic DNA that has ligated to a splinkerette oligonucleotide. In addition, background products are reduced due to the stable hairpin loop on the splinkerette: 1) it will not ligate to genomic DNA to generate non-specific priming and 2) it reduces end-repair priming [9]. Since the enzyme does not need to cut within the transgene, any restriction enzyme that produces sticky ends can be used with the appropriate splinkerette oligonucleotide. This suggests that larger genomic fragments flanking the transgene insertion site can be isolated.

We have adapted spPCR for the mapping of transposable elements (both P-elements and piggyBacs) in *Drosophila*. The spPCR protocol we present is simple, efficient, and highly effective. To date, every transgene we have attempted to map (n>250) could be mapped by spPCR. Splinkerette PCR could be applied to the mapping of transgenes which were impossible using iPCR or plasmid rescue. To demonstrate the utility of spPCR, we have mapped the insertion sites for enhancer traps located within natural transposons, one of which highlights a master regulatory region for expression in a population of olfactory neurons. We have also mapped all the commonly used centromeric FRT insertion sites, some of which were within natural transposons. The spPCR protocol can be further extended for the mapping of any transgene in the *Drosophila* genome.

## Materials and Methods

### Splinkerette PCR

Details for performing spPCR for P-element and piggyBac elements can be found in the spPCR protocol, Splinkerette Protocol S1. For PCR amplifications, Phusion Taq polymerase (Finnzymes) was used. In a spPCR reaction, the size of non-genomic DNA (*i.e.*, P-element specific DNA) amplified when mapping GAL4 enhancer traps is 279 bp for the 5′P end and 43 bp for the 3′P end. For all other P-elements, the size of non-genomic DNA in a PCR reaction is 111 bp for the 5′P end and 43 bp for the 3′P end. Subtracting these numbers from the PCR fragment sizes indicate the extent of the isolated flanking genomic DNA.

### Inverse PCR

Purified genomic DNA (∼1 µg; QIAGEN DNeasy kit) was digested by BfuCI (NEB) for 8 h. Digested DNA (∼0.5 µg) was self ligated (T4 DNA Ligase, NEB) for 2 h at 25°C in a total volume of 50 µl. For isolating 5′ P-element insertion sequence, primer pairs PGAW2 (CAGATAGATTGGCTTCAGTGGAGACTG) and PGAW3 (CGCATGCTTGTTCGATAGAAGAC) were used. For isolating 3′ P-element insertion sequence, primer pairs PRY4 (ACTGTGCGTTAGGTCCTGTTCGTT) and PRY1 (CCTTAGCATGTCCGTGGGGTTTGAAT) were used. iPCR products were sequenced with Sp1 (ACACAACCTTTCCTCTCAACAA; 5′ insertion sites) or Spep1 (GACACTCAGAATACTATTC; 3′ insertion sites). The PCR protocol for 5′ iPCR was 98°C 75 sec, 35 cycles of 98°C 30 sec, 65.5°C 30 sec, 72°C 2 min, followed by 72°C 7 min. For 3′ iPCR the PCR protocol was 98°C 75 sec, 35 cycles of 98°C 30 sec, 62.5°C 30 sec, 72°C 2 min followed by 72°C 7 min. Phusion Taq (NEB) was used for all PCR reactions.

In an iPCR reaction, the size of non-genomic DNA (*i.e.*, P-element specific DNA) amplified when mapping GAL4 enhancer traps is 553 bp for the 5′P end and 243 bp for the 3′P end. For all other P-elements, the size of non-genomic DNA in a PCR reaction is 1218 bp for the 5′P end and 243 bp for the 3′P end. Subtracting these numbers from the PCR fragment sizes indicate the extent of the isolated flanking genomic DNA.

### Transgenic animal construction

#### GH146-GAL4 transgene

The cloning of the GH146-GAL4 enhancer region and generation of GH146-GAL4 transgenic animals were described in [11].

#### NP225-GAL4 transgene

**Figure 2:**
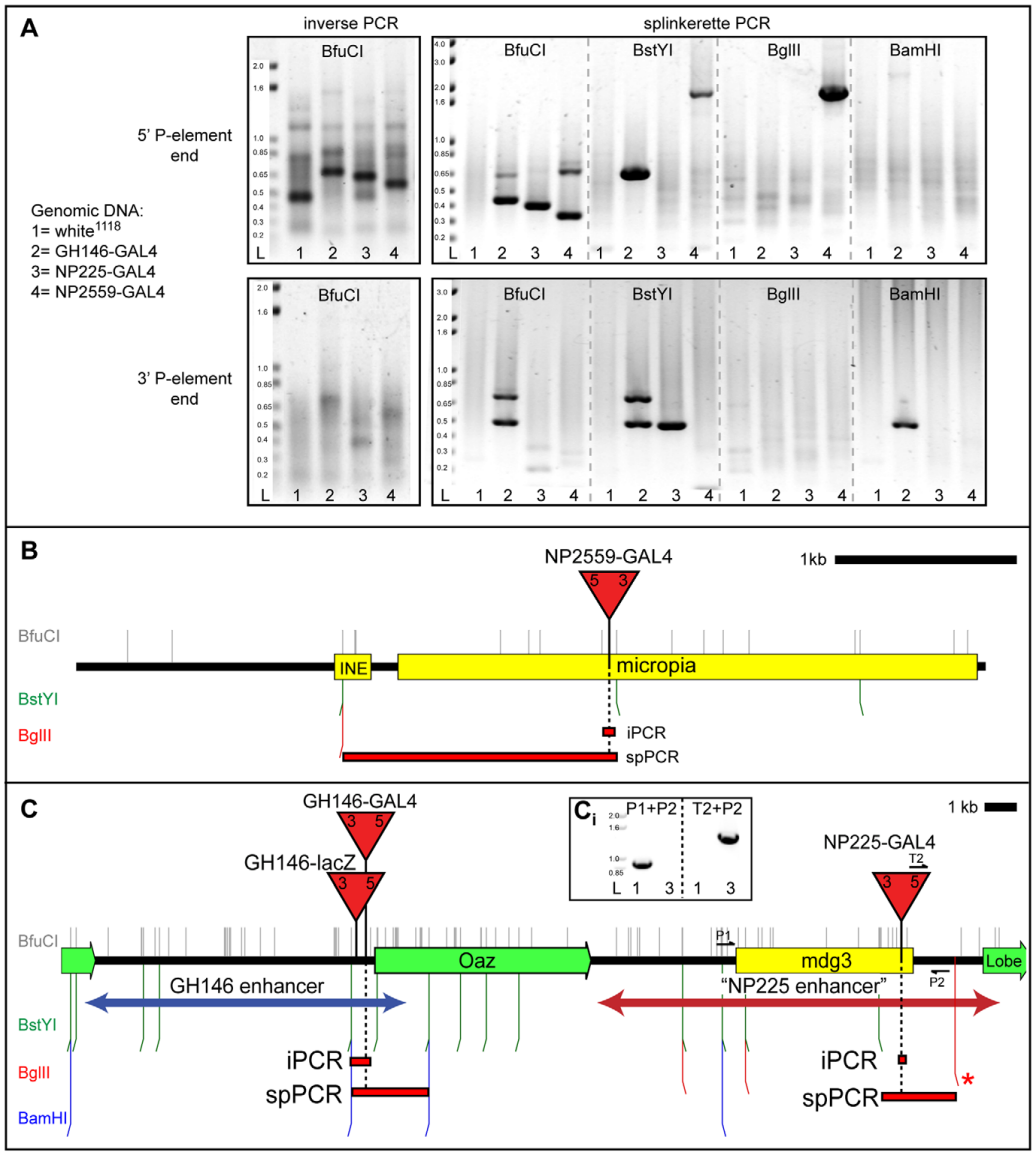
Comparison of spPCR and iPCR for mapping of P-elements in *Drosophila*. **A**) Representative agarose gels showing 5 µl of PCR products for inverse PCR and spPCR reactions. Genomic DNA from four fly strains (*white^1118^* serves as a negative control) were subjected to iPCR or spPCR to isolate the 5′ or 3′ flanking genomic DNA of the P-element insertion site. For spPCR, genomic DNA was digested separately with four restriction enzymes (BfuCI, BstY1, BglII, BamHI) which produce GATC sticky ends compatible with the spPCR protocol. BfuCI iPCR products are larger than BfuCI spPCR products since iPCR amplifies more P-element specific (non-flanking genomic) DNA. DNA ladder (L) units are in kB. **B**) Schematic of the genomic locus containing the mapped NP2559-GAL4 enhancer trap element within the *micropia* natural transposon. **C**) Schematic of the genomic loci for the mapped GH146-GAL4 and NP225-GAL4 enhancer trap elements. The cloned GH146 and NP225 enhancer regions are shown as double-headed arrows. The PCR products for the NP225-GAL4 5′P-element BstYI and BglII spPCR fragments could not be seen on an agarose gel, but reliable sequence was obtained after phosphatase/exonuclease I treatment of the PCR product (see Splinkerette Protocol S1 for details). The flanking BglII site (marked by a *) is predicted based on the largest size (∼800 bp) of sequenced spPCR products. **Ci**) Agarose gel showing PCR products from the diagramed primer pairs. The P-element specific T2 primer is also diagramed in Figure 1. The lanes are labeled as in A. The *mdg3* transposon at this location is not in the *white^1118^* strain. Red triangles represent P-elements (not drawn to scale). Location of restriction sites are diagramed as vertical lines color coded according to the restriction enzymes. Restriction sites within the P-element are not shown. Black bars represent genomic DNA, green bars represent genes, yellow bars represent natural transposons, and red bars represent the extent of the longest amplified iPCR or spPCR genomic DNA fragment flanking the P-element insertion site.

NP225-GAL4 is located within a previously unmapped *mdg3* natural transposon in the genomic region 5′ to the *Lobe* gene (Figure 2C). The presence (6.4 kb band) or absence (876 bp band) of this *mdg3* transposon in different fly strains was determine using PCR primer pairs P1(TCGAGCGTGTTTATGCTTTG) and P2 (TTGTCACACTCTGAGGCCAG) (see Figure 2C_i_). This *mdg3* natural transposon was also found in NP line NP80-GAL4, but it is not in GH146-GAL4 or *white^1118^*. FlyBase (GenomeBrowser, R5.19) indicates that there is a *roo* natural transposon in the 3′ region of the *Oaz* gene. However, this *roo* natural transposon is not present in NP225-GAL4 or NP80-GAL4 genomic DNA, as determined by genomic PCR analysis (data not shown).

The genomic region corresponding with the NP225-GAL4 insert, including the *mdg3* natural transposon (see Figure 2B), was PCR amplified from NP80-GAL4 genomic DNA (which also contains the mdg3 element in this location) using NP225regionFOR-CACC(CACCTGATAGTTTTTCAAAGATTCGACTTCGCTG) and NP225regionREV(CGGAGACAGTCGACAAAAAAATTGAACG) primers. This gives a band of approximately 8 kb which was cloned into the pENTR-TOPO cloning vector (Invitrogen). This NP225 genomic region was then shuttled into the Phi-C31 attB pBPGUw vector [12] using the Gateway LR Clonase II Enzyme kit (Invitrogen). This places the enhancer region 5′ to the *Drosophila* synthetic core promoter and GAL4 coding region. The construct was integrated into two attP landing sites, attP2 [13], [14] and attP86Fb [15] by Phi-C31 integrase-mediated transformation [13].

The NP225-GAL4 insertion site was confirmed (Figure 2C_i_) using primer pairs T2 (5′SPLNK-GAWB#2, GAGCTTTTTAAGTCGGCAAATATCG) and P2.

#### Immunohistochemistry

Confocal images were taken on a LSM 510 Confocal Microscope (Zeiss). The procedures for fixation, immunochemistry and imaging were as described previously [16]. Primary antibodies used were Rat anti-CD8 (Caltag Laboratories, 1∶200), Mouse anti-nc82 (DSHB, 1∶25), and Rabbit anti-β-galactosidase (1∶100).

## Results

### General strategy

The spPCR method requires a sticky end generated by a Class II restriction endonuclease with 5′ overhanging nucleotides [9]. To allow for a wide range of fragment sizes, we chose a GATC overhang for our splinkerette oligonucleotide design (Figure 1C; Splinkerette Protocol S1). This allows for the use of 4 restriction enzymes that will yield compatible GATC sticky ends- BfuC1 (↓GATC), BstYI (R↓GATCY), BglII (A↓GATCT), and BamHI (G↓GATCC) which cut with decreasing frequency in the *Drosophila* genome (see Splinkerette Protocol S1). We reasoned that this degree of flexibility in restriction rates should allow for essentially any size fragment to be isolated. For example, if small fragments are required, then digesting with BfuCI or BstYI could be performed. If larger flanking genomic DNA were required (for example, to extend beyond a natural transposon), then BglII or BamHI could be used. We have validated and optimized a set of oligonucleotide primers for the isolation of both 5′ and 3′ flanking genomic DNA for P-element and piggyBac transposons (Splinkerette Protocol S1 and Table 1). Most significantly, since the 3′ P-element primer set was designed to match the P-element's 3P transposon end, the same primer set can be used to isolate the 3′ flanking genomic DNA of any P-element, regardless of internal transgenic components. As such, mapping by spPCR is much simpler compared to iPCR since one set of conditions can be used to map any P-element. To date, we have successfully used spPCR to map all transposable elements (P-element and piggyBac) we have attempted (n>250; Figure 2 and data not shown). This high success rate is possible due to the ability of spPCR to isolate longer and longer flanking genomic regions (by using rarer and rarer genomic restriction sites) until a unique BLAST score is achieved. To facilitate the use of spPCR for the mapping of transposable elements in *Drosophila*, we have generated a simple and effective protocol for use by the *Drosophila* community (Splinkerette Protocol S1).

**Table 1:** Oligonucleotides for Splinkerette PCR of *Drosophila* P-elements^1^.

Oligonucleotide Name	Oligonucleotide Sequence	Purpose
SPLNK-GATC-TOP^2^	**GATC**CCACTAGTGTCGACACCAGTCTCTAATTTTTTTTTTCAAAAAAA	Top strand of splinkerette oligonucleotide with **GATC** sticky end
SPLNK-BOT^2^	CGAAGAGTAACCGTTGCTAGGAGAGACCGTGGCTGAATGAGACTGGTGTCGACACTAGTGG	Bottom strand of splinkerette oligonucleotide
SPLNK#1^2^	CGAAGAGTAACCGTTGCTAGGAGAGACC	Splinkerette specific primer for Round 1 PCR
SPLNK#2^2^	GTGGCTGAATGAGACTGGTGTCGAC	Splinkerette specific primer for Round 2 PCR
3′SPLNK#1	CACTCAGACTCAATACGACAC	Round 1 PCR primer for 3′ end of all P-elements
3′SPLNK#2	GGATGTCTCTTGCCGAC	Round 2 PCR primer for 3′ end of all P-elements
3′SPLNK-SEQ	CGGGACCACCTTATG	Sequencing primer for all 3′ end spPCR reactions
5′SPLNK#1-CASPR	ATAGCACACTTCGGCACG	Round 1 PCR primer for 5′ end of pCaSpeR based P-elements^3^
5′SPLNK#2-CASPR	ATTCGTCCGCACACAACC	Round 2 PCR primer for 5′ end of pCaSpeR based P-elements^3^
5′SPLNK-CASPR-SEQ	CCTCTCAACAAGCAAACG	Sequencing primer for 5′ end pCaSpeR PCR reactions
5′SPLNK#1-GAWB	TGGGAGAGTAGCGACACTCC	Round 1 PCR primer for 5′ end of GAL4 enhancer trap P-elements
5′SPLNK#2-GAWB	GAGCTTTTTAAGTCGGCAAATATCG	Round 2 PCR primer for 5′ end of GAL4 enhancer trap P-elements
5′SPLNK-GAWB-SEQ	CTCAACAAGCAAACGTGC	Sequencing primer for 5′ end of GAL4 enhancer trap PCR reactions

1 See Splinkerette Protocol S1 for spPCR conditions for piggyBac elements.

2 Splinkerette oligonucleotide sequences from [10].

3 See Splinkerette Protocol S1 for list of compatible pCaSpeR based P-elements.

### Mapping enhancer traps inside natural transposons—identification of a master regulatory region of olfactory neuronal expression

Enhancer traps are among the most useful transgenic lines in *Drosophila* [17], [18], [19]. Besides being a useful tool to drive effector transgenes in tissue specific patterns, enhancer traps also highlight important regulatory elements in the *Drosophila* genome. We are interested in GAL4 enhancer traps (and their regulatory elements) that label a particular neuronal population in the brain, the olfactory projection neurons (PNs). There are ∼60 different types of PNs that target dendrites to ∼54 discrete foci called glomeruli in the main olfactory organizing center, the antennal lobe. PNs send axons to higher olfactory processing centers in the brain (the mushroom bodies and lateral horn). Our initial attempts to map by iPCR many of our PN expressing GAL4 enhancer traps failed due to their locations in natural transposons (Figure 2 and data not shown). In contrast, we used spPCR to successfully map all of these GAL4 enhancer traps. Figure 2 provides an example for using spPCR to map two such enhancer trap insertions.

The NP2559-GAL4 enhancer trap is inserted into a *micropia* natural transposon, and could not be mapped by iPCR (Figure 2A, 2B). However, by using spPCR and digestion with BstYI or BglII, genomic DNA flanking the 5′P-element end could be isolated that extended beyond this natural transposon and into unique genomic sequences (Figure 2A, 2B, Table S1).

**Figure 3:**
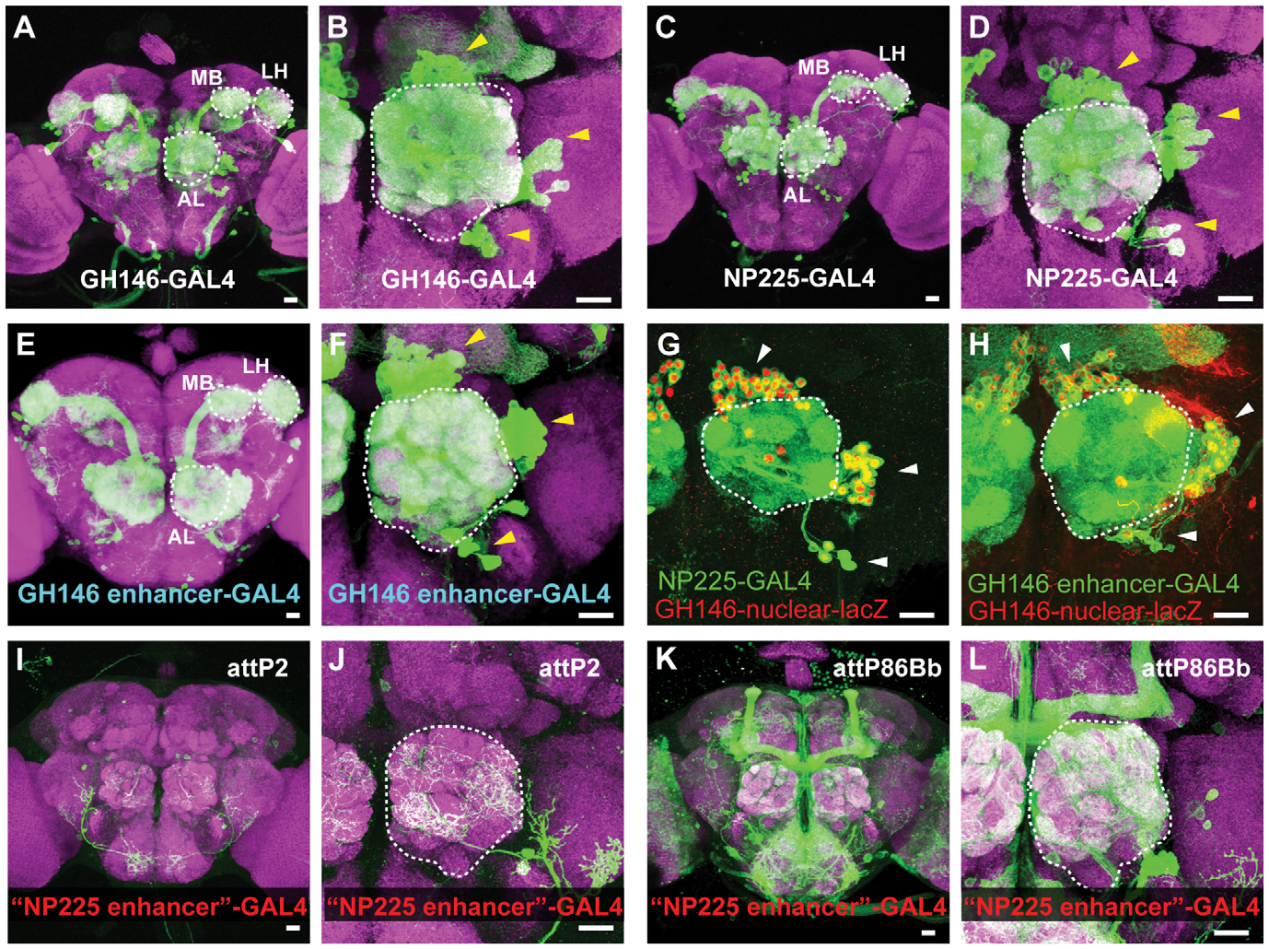
Splinkerette mapping of an enhancer trap within a natural transposon highlights a master regulatory region for PN expression. **A**) The expression pattern of the GH146-GAL4 enhancer trap in a representative confocal projection of a whole mount *Drosophila* brain immunostained for a general neuropil marker (monoclonal antibody nc82) in magenta, and for mCD8 in green (which detects GAL4-dependent UAS-mCD8-GFP expression). The antennal lobe (AL), mushroom body calyx (MB) and lateral horn (LH) regions are outlined. **B**) A higher magnification of the antennal lobe region for the GH146-GAL4 expression pattern. Arrowheads point to the three clusters (dorsal, lateral, ventral) of cell bodies of the projection neurons (PNs). The antennal lobe is outlined. **C**) The expression pattern of the NP225-GAL4 enhancer trap in whole mount brain confocal projections. **D**) A higher magnification of the antennal lobe region for the NP225-GAL4 expression pattern. **E–F**) The representative expression pattern of a transgenic construct that drives GAL4 expression from the cloned GH146 enhancer region diagramed in Figure 2C. The expression pattern in PNs appears identical to the GH146-GAL4 enhancer trap line. **G**) Representative confocal projections of the antennal lobe of GH146-lacZ and NP225-GAL4 animals immunostained for ßgal (in red) and for mCD8 (which reports GAL4-dependent UAS-mCD8-GFP expression) in green. **H**) Confocal projection of the antennal lobe of GH146-lacZ and transgenic GH146-GAL4 animals. **I–J**) The expression pattern of transgenic flies that contain the genomic DNA near the NP225 insertion site (“NP225 enhancer”) driving GAL4 integrated into the attP2 Φ-C31 genomic site. **K–L**) The expression pattern of transgenic flies that contain the “NP225 enhancer” region driving GAL4 integrated into the attP86Bb Φ-C31 genomic site. Expression in the antennal lobe is from innervation from olfactory receptor neurons, and not PNs. Scale bars: 20 µm.

The mapping of enhancer traps within natural transposons led to a particularly interesting result while investigating the genomic regulatory region corresponding to the GH146-GAL4 enhancer trap. The GH146-GAL4 enhancer trap [20], [21] labels most of the PN classes in the *Drosophila* brain (Figure 3A, 3B). The insertion site for GH146-GAL4 maps by iPCR and spPCR to the promoter region of the *oaz* gene on chromosome arm 2R (Figure 2A, 2C) [22]. This suggests that this genomic region contains the regulatory elements required to specify expression in this particular neuronal population. Indeed, a lacZ enhancer trap in this location (GH146-lacZ) also expresses in the same PN population (data not shown). To determine if this genomic region is sufficient to induce expression in PNs, and thus contains the regulatory DNA required for this expression, a transgenic construct was generated which used this genomic region to drive the GAL4 transcription factor (Figure 2C). Transgenic flies, in which this transgene was inserted at a different location in the genome, was sufficient to drive the same expression pattern as the GH146-GAL4 and GH146-lacZ enhancer traps (Figure 3E, 3F). This indicates that this genomic region contains all the regulatory elements sufficient to reproduce the expression pattern of the native GH146-GAL4 enhancer trap.

In a screen of thousands of GAL4 enhancer traps, additional enhancer traps were identified that gave rise to very similar GH146-GAL4 expression patterns [23]. All of these GAL4 enhancer traps were mapped by iPCR to the same genomic locus as GH146-GAL4 (*e.g*, NP5288-GAL4, data not shown) except for one: NP225-GAL4. The expression pattern of this enhancer trap is essentially identical to GH146-GAL4 ([23], also Figure 3C, 3D) except that NP225-GAL4 does not label the anterior paired lateral neuron which innervates the mushroom body lobes [24]. Nonetheless, NP225-GAL4 labels the same set of PNs as GH146-GAL4 and GH146-lacZ (Figure 3G). The NP225-GAL4 insertion site could not be molecularly mapped by plasmid rescue (data not shown) or by iPCR because it is within a *mdg3* natural transposon (Figure 2A, 2C; Gal4 Enhancer Trap Insertion Database, Kyoto, Japan).

We were interested in determining the genomic locus responsible for the expression pattern of NP225-GAL4. Does it represent a new location in the *Drosophila* genome that can regulate precise expression in this PN population? If so, how is this genomic region structurally related to the GH146-GAL4 enhancer region? To address these questions, we used spPCR to isolate flanking genomic sequences that extended beyond the *mdg3* natural transposon (Table S1). Surprisingly, NP225-GAL4 mapped to a region 16 kb away from the GH146-GAL4 insertion site, 3′ to the oaz gene (Figure 2C). This genomic region was not predicted by the Berkeley Drosophila Genome Project (R5.19) to contain a *mdg3* natural transposon, and hence represents a divergence between the sequenced strain and the strain used for the GAL4 enhancer trap screen.

To determine if this genomic region was also sufficient to drive expression in PNs, we generated a GAL4 transgenic construct driven by the putative NP225-GAL4 enhancer region surrounding the transposon insertion site (Figure 2C), and integrated it into two different genomic locations (Figures 3I, 3J, 3K, 3L). Neither of these transgenic lines expressed in the PN population, but did show great variability in their expression patterns. This indicates that the genomic region near the insertion site of NP225-GAL4, when removed from its original genomic locus, cannot induce expression in PNs (the innervation in the antennal lobe in Figure 3K and 3L is from olfactory receptor neuron innervation). The great variability in the expression patterns between the two insertion sites further suggests that the NP225 genomic region is highly susceptible to position effects, perhaps by presence of a natural transposon [25], and can be influenced by the regulatory elements of neighboring genomic elements. As such, it is likely that the NP225-GAL4 enhancer trap has co-opted the regulatory elements in the genomic region defined by GH146-GAL4 to direct its expression pattern. Since every enhancer trap identified to date that expresses in this particular neuronal PN pattern localizes to this genomic locus suggests that this genomic region is a master regulatory region for PN expression. It will be interesting to further dissect how this genomic region can lead to expression in a diverse set of PNs.

### Mapping difficult insertions sites—the centromeric FRT insertions

Since centromeric regions are more likely to contain natural transposons, the mapping of transgenes inserted into this region would be an ideal test for the efficacy of the spPCR method. A set of commonly used transgenes inserted into the centromeric region are P-elements that contain FRT recombination sites [26], [27]. Chromosomes that contain FRT sites can be induced to undergo mitotic recombination, and hence are widely used for performing mosaic analysis in *Drosophila*. These FRT containing P-elements were originally mapped by *in situ* hybridization to polytene chromosomes which can localize the insertion to a polytene band (on the order of approximately 150,000 bp). Molecularly mapping the exact FRT insertion site will also precisely define which genes are accessible for mosaic analysis.

All FRT insertion sites were successfully mapped by spPCR (Table 2, Table S1). As expected, some of the FRT containing P-elements were inserted into natural transposons, and likely could not have been mapped by iPCR or plasmid rescue. The P{neoFRT}80B insertion is located within a *1360* natural transposon. Mapping the P{neoFRT}19A insertion was a particularly good test for spPCR: it is located within a natural transposon (*jockey*) which itself is directly adjacent to another natural transposon (*Rtc1*). Nonetheless, by performing spPCR using BamHI as the restriction enzyme, flanking genomic DNA could be isolated that extended beyond these natural transposons. Also of note, P{neoFRT}42D is inserted into the first exon of the *coronin* gene. The FRT42D insertion might affect the function of this gene (which is predicted to be involved in regulating the actin cytoskeleton [28]), and caution should be used when performing mosaic analyses using this FRT insertion. As an alternative, P{FRT(whs)}G13 could be used, which is inserted more centromeric (at 42B1) than FRT42D (at 42D6). A disadvantage of using FRTG13 is that it is marked by the *white+* transgene which leads to dark red eyes in a *white* mutant background. This dark red eye can be inconvenient when trying to identify FRTG13 recombinants with additional transgenes that are also marked by *white+*. To circumvent this problem, we have validated PCR primers that can be used to specifically test for the insertion of FRTG13 (as well as the other FRT insertions) (Table 3).

**Table 2:** Splinkerette PCR mapping of commonly used FRT insertions.

FRT Name^1^	Previous Map Position^2^	Splinkerette Map Position	Insertion Site^3^	Inside natural transposon	Insertion Notes^4^
P{neoFRT}19A	19A	19A2	X:19804903	jockey and Rtc1	Repetitive region
P{neoFRT}40A	40A	40A3	2L:21794705		1^st^ intron of *CG31612*
P{FRT(w^hs^)}G13	42B	42B1	2R:2389386		5′ region of *jing*
P{neoFRT}42D	42D	42D6	2R:2760212		Inside 1^st^ exon of *coro*
P{FRT(w^hs^)}2A	79D-F	80B1	3L:22865175		5′ region of *Arf79f*
P{neoFRT}80B	80B	80D1	3L:23096809	1360{3899}	3′ region of *CkII alpha*
P{neoFRT}82B	82B	82B2	3R:278974		5′ region of *CG31522*

1 P{neoFRT} from [27]. P{FRT(w^hs^)} from [26], [27], [28].

2 Based on polytene chromosome *in situ* hybridization [26], [27], [28].

3 BDGP version R5.14.

4 Sequence results of spPCR reactions provided in Table S1.

**Table 3:** PCR conditions to test for FRT insertions.

For Checking:	Primer Pair:	Phusion Taq annealing Tm	Size
40A FRT	40A-Specific-FOR 3′SPLNK#1	59°C	539 bp
40A FRT	40A-Specific-REV 5′SPLNK#2-CASPR	62°C	275 bp
82B FRT	82B-REV#1 5′SPLNK#2-CASPR	62°C	700 bp
G13 FRT	G13-SPECIFIC-FOR 3′SPLNK#1	59°C	278 bp
G13 FRT	G13-SPECIFIC-REV 5′SPLK#2-CASPR	62°C	319 bp
2A FRT	2A-SPECIFIC-FOR 3′SPLNK#1	59°C	564 bp
2A FRT	2A-SPECIFIC-REV 5′SPLK#2-CASPR	62°C	384 bp

Primer Name	Primer Sequence
3′SPLNK#1	CACTCAGACTCAATACGACAC
5′SPLNK#2-CASPR	ATTCGTCCGCACACAACC
40A-Specific-FOR	GTGGGCTCGGCAACATTCTG
40A-Specific-REV	TCGAGACCCTCATCCGAACG
82B-REV#1	AATGCCGTCACCTACACACG
G13-SPECIFIC-FOR	CCGCTCTCGAAAACCTGCTG
G13-SPECIFIC-REV	AAACGCCATCGATTGGCAAG
2A-SPECIFIC-FOR	GCCAAACACACCACACACCG
2A-SPECIFIC-REV	CAACAGCGGAGCAAGTGCAG

## Discussion

Splinkerette PCR is a powerful method for isolating the genomic regions flanking known sequences. We have applied spPCR for mapping of P-elements and piggyBac elements, but it can be easily adapted for the mapping of viral integration sites or of additional transposable elements carrying transgenes, such as *minos* [29], *hobo* [30], *mariner* [31], or *Hermes* [32]. We have also shown that even the most difficult insertion sites can be mapped using spPCR. As such, transposon insertions for all GAL4 enhancer traps which are currently unmappable by iPCR could potentially be mapped by spPCR. In a large scale enhancer trap screen, 6966 insertions were analyzed by iPCR. Of those, 2536 (36%) could not be mapped by iPCR due to small flanking sequence or insertion into repetitive sequence or natural transposons (GETDB, Kyoto, Japan). Similarly, in a large scale piggyBac gene disruption screen, 11% of insertions could not be mapped by iPCR [33]. Splinkerette PCR could be applied for the mapping of these insertion sites.

In our spPCR protocol, we targeted GATC sticky ends for the isolation of flanking genomic DNA. This allows for use of a number of different restriction enzymes that cut genomic DNA with different frequencies, and hence generate a predictable range of flanking genomic DNA that could be isolated. Importantly, by modifying only the TOP strand of the splinkerette oligonucleotide, different sticky ends could be targeted (see Figure 1C). For example, replacing GATC with AATT will target the splinkerette to sites generated by EcoRI (G↓AATTC) digestion, and replacing GATC with GGCC will target the splinkerette to sites generated by NotI (GC↓GGCCGG) digestion. Few other changes to the spPCR protocol (besides enzyme choices) would be required. This would extend the flexibility of the spPCR method to isolate different flanking genomic segments not targeted by our current protocol.

Splinkerette PCR is also simpler to set up than iPCR. For example, mapping the 3′ end of the P-element transgenes PZ, PlacW, PGAWB, and PEP by iPCR each require a specific set of primers for the PCR reaction, and another set of primers for the sequence reaction, which in turn depends on which enzyme was used for the restriction digest (http://www.fruitfly.org/about/methods/inverse.pcr.html). In contrast, the 3′ end of all P-elements can be mapped by using the same splinkerette primers and conditions. Given the high success rate of spPCR and its ease of use, it could be applied for standard mapping of transgenes, or for high-throughput screens.

The spPCR conditions described here can be applied to most P-elements, even if the internal components of the P-element vector are unknown. This also applies to naturally occurring P-elements. While performing control experiments using the CASPR set of spPCR primers, we made the startling discovery that our *white^1118^* stock contained a KP element inserted on the third chromosome (data not shown). KP elements are naturally occurring P-elements that contain the same 5′ and 3′ P-element ends as the pCaSpeR based constructs, but do not contain a visible marker or a transposase gene [34], [35]. And although they cannot mobilize without the addition of exogenous transposase, if they are presented with transposase, their mobilization might cause mutations that could go unnoticed if no dramatic defects in viability or sterility result. As such, spPCR could be used to test for the presence of unexpected P-elements in one's lab stocks, especially if those stocks will be used for behavior or for transformation of transgenic constructs. Of note, the isogenized *white* stocks from Bloomington Stock Center (Stock Numbers 5905 and 6326) and the Canton-S strain (Stock number 1) do not contain P-elements as determined by spPCR (data not shown).

Splinkerette PCR is similar in design to adapter-ligation PCR used in *Arabidopsis* to map T-DNA insertions [36]. In this technique, an annealed double stranded oligonucleotide is also ligated to digested genomic DNA. The major difference between spPCR and adapter-ligation PCR is the design of the annealing oligonucleotide: spPCR uses unmodified oligonucleotides that form a hairpin loop to reduce unwanted PCR amplifications, whereas adapter-ligation PCR used phosphorylated and C7 amino modified oligonucleotides for such a purpose. The advantage of adapter-ligation PCR is that only a single round of PCR is required, whereas spPCR often requires two rounds of nested PCR. Given the success of spPCR in the *Drosophila* system, adapter-ligation PCR might also be adaptable for mapping of transposable elements. Also of note, thermal asymmetric interlaced-PCR (TAIL-PCR) has been successfully used to map many P-element and piggyBac insertions [37]. However, this protocol requires three rounds of nested PCR using three different degenerate primers. Since spPCR uses unmodified oligonucleotides, it might be more cost effective when initially trying to map difficult transposon insertions.

The splinkerette protocol described here has also been streamlined and simplified from previously described mammalian protocols [9],[10],[38]. For example, we found that ligation of the splinkerette to digested genomic DNA can be shortened to two hours at room temperature (as opposed to overnight incubations at 4°C or 16°C), and that column purification of the splinkerette ligation reaction was not necessary. As a result, splinkerette mapping can easily be performed in one to two days with reduced expense. Such changes might also be applicable to the splinkerette protocols used in mammalian systems.

## Supporting Information

Table S1 Splinkerette PCR sequences for FRT insertion sites, GH146-GAL4, NP225-GAL4, and NP2559-GAL4.
(0.03 MB XLS)

Splinkerette Protocol S1 A detailed step-by-step protocol for performing spPCR to isolate the flanking genomic DNA of P-element and piggyBac insertions.
(0.15 MB DOC)
