# Supplementary material for: Splinkerette PCR for Mapping Transposable Elements in Drosophila
Source: PLoS One. 2010 Apr 13;5(4):e10168. doi: 10.1371/journal.pone.0010168 (PMC2854151; doi:10.1371/journal.pone.0010168)
Supplement: Splinkerette Protocol S1 — A detailed step-by-step protocol for performing spPCR to isolate the flanking genomic DNA of P-element and piggyBac insertions. (0.15 MB DOC) [file pone.0010168.s002.doc]

**Splinkerette PCR Protocol for**

**Mapping Transposable Elements in *Drosophila***

Potter and Luo

**Protocol Outline**

1) Genomic Prep (30 mins)

2) Genomic Digest (2 hrs → O/N)

3) Ligation of digested genomic DNA to annealed splinkerette oligonucleotide (2 hrs)

4) Round 1 PCR (2 hrs)

5) Round 2 PCR (1.5 hrs)

6a) Ant Phos/ExoI treat Round 2 DNA (2hrs)

6b) or Run Round 2 PCR on gel and purify band (1 hr)

7) Sequence

***Step 1. Genomic Prep***

There are many established methods for genomic DNA preparations. When performing splinkerette PCR on a small number of lines, we recommend the QIAGEN DNeasy kit (Qiagen Inc., Valencia, CA). Elute in 200 µl AE buffer. Typical DNA concentrations are ~ 20-50 ng/µl.

For many samples (*e.g.,* 96 well plates) follow the genomic preparation protocol established in [1]. The splinkerette protocol also works with a simple genomic prep from single flies (http://engels.genetics.wisc.edu/flyDNA.html).

***Step 2. Genomic Digest***

**Table 1. Genomic Digest Reaction.**

| **Components** | **Volume** |
| --- | --- |
| genomic DNA (~20-40 ng/µl) | 25 µl |
| H_2_O | 2 µl |
| 10X BSA | 3.5 µl |
| 10X NEB BUFFER | 3.5 µl |
| ENZYME | 1 µl |
| **Total** | **35 µl** |

**Table 2. Restriction Enzyme Conditions (to generate GATC sticky ends).**

| **Enzyme** | **Cut Site** | **Digest Temp** | **Heat inactivate?** | **Purify digest?** |
| --- | --- | --- | --- | --- |
| BamH1 | G↓GATC C | 37˚C | No | yes |
| BglII | A↓GATC T | 37˚C | No | no |
| **BstYI** | R↓GATC Y | 60˚C | Yes, 20 min @80˚C | no |
| BfuCI | ↓GATC | 37˚C | Yes, 20 min @80˚C | no |

2.1 See Table 12 for details on the compatible restriction enzymes. Digest genomic DNA ≥ 2 hrs. As listed in Table 2, heat inactivate or purify the enzymatic reaction if required. This is necessary in cases where the splinkerette oligonucleotide ligated to genomic DNA regenerates the restriction site.

*Note*: We recommend first using **BstYI**. This enzyme is often sufficient for mapping most insertions. Longer DNA fragments can be isolated by using BglII or BamHI as needed. When doing splinkerette PCR on a few samples, we also recommend performing splinkerette PCR on both the 5’ and 3’ ends of the transposable element. Each reaction should indicate the same genomic insertion site. If not, this is a good indicator that the fly line has multiple transposon insertions. Also, it is highly recommended to perform splinkerette PCR on a negative control genomic sample that does not contain P-elements, such as *white^1118^* or Canton-S flies. This will verify that there are no contaminants in the reagents, as well as confirm that wild-type stocks are indeed free of P-elements.

***Step 3. Ligation to Splinkerette Oligonucleotide***

Table 3. Conditions for Ligating Digested Genomic DNA to Annealed Splinkerette Oligonucleotide

| **Components** | **Volume** |
| --- | --- |
| Digested genomic DNA | 35 µl |
| H_2_O | 2.5 µl |
| 10X NEB Ligase Buffer | 5 µl |
| annealed splinkerette oligonucleotide (see Table 14) | 6 µl |
| NEB T4 DNA Ligase (400U/µl) | 1.5 µl |
| **Total** | **50 µl** |

3.1 Incubate at room temperature ≥ 2 hrs. Proceed directly to Round 1 PCR.

*Note*: ligation reactions can be incubated at 16ºC for 16 hrs, but an increase in efficacy was not found.

***Step 4. Round 1 Splinkerette PCR***

**Table 4. Round 1 PCR Reaction.**

| **Components** | **Volume** |
| --- | --- |
| Ligated genomic DNA | 10 µl |
| H_2_O | 8.25 µl |
| 5x Phusion HF Buffer | 5 µl |
| 10 mm dNTP | 0.5 µl |
| SPLNK#1,10 µM | 0.5 µl |
| Primer #1, 10 µM (see Table 5) | 0.5 µl |
| Phusion Taq (Finnzymes) | 0.25 µl |
| **Total** | **25 µl** |

**Table 5. Primer Conditions for Round 1 PCR.**

| **Purpose** | **Primer #1** | **xx anneal temp** |
| --- | --- | --- |
| 3’ end of any P-element | 3’SPLNK#1 | 61ºC |
| 5’ end- CASPER | 5’SPLNK#1-CASPR | 61ºC |
| 5’ end- GAWB (GAL4 enhancer trap) | 5’SPLNK#1-GAWB | 63.5ºC |
| 3’end – piggyBac | 3’SPLNK-PB#1 | 58ºC |
| 5’end – piggyBac | 5’SPLNK-PB#1 | 64ºC |

**Table 6. PCR conditions for ro**und 1 PCR.

| 98ºC 75 sec → | 98ºC 20 sec  64ºC 15 sec | (x 2) → | 98ºC 20 sec  xx ºC 15sec  72ºC 2 min | (x 30) → | 72ºC 7 min → | 4ºC hold |
| --- | --- | --- | --- | --- | --- | --- |

4.1 Perform Round 1 PCR as detailed in Tables 4-6. We recommend using NEB Phusion Taq (Finnzymes) as this gave us the longest PCR fragments. PCR conditions have been optimized for Phusion Taq.

***Step 5. Round 2 Splinkerette PCR***

**Table 7. Round 2 PCR Reaction.**

| **Components** | **Volume** |
| --- | --- |
| Round 1 PCR | ≤1 µl |
| H_2_O | 17.25 µl |
| 5x Phusion HF Buffer | 5µl |
| 10 mm dNTP | 0.5 µl |
| SPLNK#2 ,10 µM | 0.5 µl |
| Primer #2, 10 µM (see Table 8) | 0.5 µl |
| Phusion Taq | 0.25 µl |
| **Total** | **25 µl** |

**Table 8. Primer Conditions for Round 2 PCR.**

| **Purpose** | **Primer #2** | **xx anneal temp** |
| --- | --- | --- |
| 3’end of any P-element | 3’SPLNK#2 | 58ºC |
| 5’ end- CASPER | 5’SPLNK#2-CASPR | 62ºC |
| 5’ end- GAWB (GAL4 enhancer trap) | 5’SPLNK#2-GAWB | 66ºC |
| 3’end - piggyBac | 3’SPLNK-PB#2 | 59ºC |
| 5’end - piggyBac | 5’SPLNK-PB#2 | 66ºC |

Table 9. PCR Conditions for Round 2 PCR.

| 98ºC 75 sec → | 98ºC 20 sec  xx ºC 15 sec  72ºC 90 sec | (x 30) → | 72ºC 7 min → | 4ºC hold |
| --- | --- | --- | --- | --- |

5.1 Perform Round 2 PCR as detailed in Tables 7-9. Best results are often obtained when using 0.5 µl round 1 PCR as template.

5.2 Analyze 5µl of Round 2 PCR on a 0.7% agarose gel. PCR band sizes will depend on which enzyme was used. Usually, BglII reactions yield ~1.5 kb bands, and BstYI reactions yield ~0.5 kb bands.

5.3 PCR bands can be gel extracted for sequence analysis. Alternatively, the remainder of the round 2 PCR reaction can be treated with Antarctic Phosphatase and Exonuclease and used directly for sequencing as detailed below. Good sequence runs can still be obtained after Step 6 even if only very weak bands are present on the gel.

***Step 6. Antarctic Phosphatase/Exonuclease I treatment***

**Table 10. AntPho/ExoI Reaction Conditions.**

| **Components** | **Volume** |
| --- | --- |
| Round 2 splinkerette PCR | 20 µl |
| 10X NEB AP Buffer | 3.0 µl |
| H_2_O | 3.0 µl |
| NEB Antarctic Phosphatase | 2.0 µl |
| NEB Exonuclease I | 2.0 µl |
| **Total** | **30 µl** |

6.1 Incubate reactions at 37ºC for 2 hrs, followed by a 80ºC incubation for 15 min. Use 15µl of the reaction for sequencing with the appropriate sequencing primer listed in Table 11.

**Table 11. Sequencing Primers**

| **Purpose** | **Primer** |
| --- | --- |
| 3’end of any P-element | 3’SPLNK-SEQ |
| 5’end-CASPER | 5’SPLNK-CASPR-SEQ |
| 5’end-GAWB | 5’SPLNK-GAWB-SEQ |
| 3’end –piggyBac | 3’SPLNK-PB-SEQ |
| 5’end –piggyBac | 5’SPLNK-PB-SEQ |

*Note*: The following splinkerette specific sequence might be at the end of a sequence reaction:

**GATC**CCACTAGTGTCGACACCAGTCTCATTCAGCCACGGTCTCTCCTAGCAACGGTTACTCTTCG

**Table 12. Restriction Enzymes with Compatible GATC Sticky Ends.**

| **Name** | **Cut Site** | **Sites/Mb in *D. melanogaster* genome^1^** | **Average fragment length in base pairs^1^** |
| --- | --- | --- | --- |
| BamH1 | G↓GATC C | 170.2 | 5874 |
| BglII | A↓GATC T | 183.3 | 5454 |
| BstYI | R↓GATC Y | 704.1 | 1420 |
| BfuCI | ↓GATC | 2758.4 | 363 |

1. http://tools.neb.com/~vincze/gnsites/comp.php?genomes=Fruit_Fly

**Table 13. Oligonucleotide Sequences**

| SPLNK-GATC-TOP | GATCCCACTAGTGTCGACACCAGTCTCTAATTTTTTTTTTCAAAAAAA |
| --- | --- |
| SPLNK-BOT | CGAAGAGTAACCGTTGCTAGGAGAGACCGTGGCTGAATGAGACTGGTGTCGACACTAGTGG |
| SPLNK#1 | CGAAGAGTAACCGTTGCTAGGAGAGACC |
| SPLNK#2 | GTGGCTGAATGAGACTGGTGTCGAC |
| 3’SPLNK#1 | CACTCAGACTCAATACGACAC |
| 3’SPLNK#2 | GGATGTCTCTTGCCGAC |
| 3’SPLNK-SEQ | CGGGACCACCTTATG |
| 5’SPLNK#1-CASPR | ATAGCACACTTCGGCACG |
| 5’SPLNK#2-CASPR | ATTCGTCCGCACACAACC |
| 5’SPLNK-CASPR-SEQ | CCTCTCAACAAGCAAACG |
| 5’SPLNK#1-GAWB | TGGGAGAGTAGCGACACTCC |
| 5’SPLNK#2-GAWB | GAGCTTTTTAAGTCGGCAAATATCG |
| 5’SPLNK-GAWB-SEQ | CTCAACAAGCAAACGTGC |
| 3’SPLNK-PB#1 | GTTTGTTGAATTTATTATTAGTATGTAAG |
| 3’SPLNK-PB#2 | CGATAAAACACATGCGTC |
| 3’SPLNK-PB-SEQ | ACGCATGATTATCTTTAAC |
| 5’SPLNK-PB#1 | ACCGCATTGACAAGCACG |
| 5’SPLNK-PB#2 | CTCCAAGCGGCGACTGAG |
| 5’SPLNK-PB-SEQ | CGACTGAGATGTCCTAAATGC |

*Note: Splinkerette oligonucleotides SPLNK-GATC-TOP, SPLNK-BOT, SPLNK#1, SPLNK#2 are derived from [2].*

**Table 14. Reaction Conditions for Annealing Splinkerette Oligonucleotides**

| **Component** | **Volume** |
| --- | --- |
| SPLNK-BOT (150 ng/µl) | 50 µl |
| SPLNK-GATC-TOP (150 ng/µl) | 50 µl |
| 10X NEB Buffer 2 | 100 µl |
| H_2_O | 800 µl |
| Total | 1000 µl |

14.1 Heat to 95ºC for 3 minutes. Allow to cool on bench to room temp (~30 mins). Store 200 µl aliquots at -20ºC.

**Table 15. P-element Constructs Compatible with 3’SPLNK:**

| *any* pCaSpeR based construct | pGT1 (BG lines) | EPgy2 (EY lines) | P{SUPorP} (KG lines) |
| --- | --- | --- | --- |
| pGaWb (GAL4 enhancer traps) | pGatB/n | pPTGAL4 | pPwl+hsGS (gene switch) |
| pUAST | pUASP | pUASdesFPc | EP |
| GS | Act5C>y+>GAL4 (Ay5C) | Flip-out constructs | ptubP-GAL80 |
| UAS-CD8GFP | pWizDir | p[acman] | pRISE |
| pICOn | pTARG | p{Switch1} | p{Switch2} |
| pP{wHy} | pFRT | pP{wlo-inGS} | pP{wlo-hsinGS} |
| pPI25.1 | *Drosophila* KP element | Dual-tagging gene trap vector pGT1 | pUChsneo (neomycin) |
| pXP (Exelixis) | pP{neoFRT} | P{FRT(whs)} | Note: This list may not be complete. |

**Table 16. P-element Constructs Compatible with 5’SPLNK-CASPR:**

| pCaSpeR | P{SUPorP} (KG lines) | EPgy2 (EY lines) | pCa4B2G |
| --- | --- | --- | --- |
| pCa4B | Ganesh-Z1 | pRISE | pUAST |
| pUASp | pXP (Exelixis) | piggyBac_PB (Exelixis) | pICON |
| p{Switch1} | p{Switch2} | pP{wHy} | RNAi cloning and transformation vector pFRiPE |
| *Drosophila* KP elements | Dual-tagging gene trap vector pGT1 | pUChsneo (neomycin) | pP{neoFRT} |
| P{FRT(whs)}-PerrimonFRT | Note: This list may not be complete. |  |  |

**5’ and 3’ piggyBac splinkerette PCR:**

Primers were based on the pXL-Bac II backbone [3]. Derivates of this vector (*e.g.*, pXL-BacII-ECFP, pXL-Bac-DsRed, pXL-BacII-SAstopDsRed [1], pBAC-GH146 [4]) will work with these splinkerette primers. These primers are also compatible with the Exelixis piggyBac vector PB and its derivatives [5].

**References**:

1. Schuldiner, O., Berdnik, D., Levy, J.M., Wu, J.S., Luginbuhl, D., Gontang, A.C. and Luo, L. (2008) piggyBac-based mosaic screen identifies a postmitotic function for cohesin in regulating developmental axon pruning. *Dev Cell*, **14**, 227-238.

2. Horn, C., Hansen, J., Schnütgen, F., Seisenberger, C., Floss, T., Irgang, M., De-Zolt, S., Wurst, W., von Melchner, H. and Noppinger, P.R. (2007) Splinkerette PCR for more efficient characterization of gene trap events. *Nat Genet*, **39**, 933-934.

3. Li, X., Harrell, R.A., Handler, A.M., Beam, T., Hennessy, K. and Fraser, M.J., Jr. (2005) piggyBac internal sequences are necessary for efficient transformation of target genomes. *Insect Mol Biol*, **14**, 17-30.

4. Berdnik, D., Fan, A.P., Potter, C.J. and Luo, L. (2008) MicroRNA processing pathway regulates olfactory neuron morphogenesis. *Curr Biol*, **18**, 1754-1759.

5. Thibault, S., Singer, M., Miyazaki, W., Milash, B., Dompe, N., Singh, C., Buchholz, R., Demsky, M., Fawcett, R., Francis-Lang, H. *et al.* (2004) A complementary transposon tool kit for Drosophila melanogaster using P and piggyBac. *Nat Genet*, **36**, 283-287.
